# The abuse potential of lemborexant, a dual orexin receptor antagonist, according to the 8 factors of the Controlled Substances Act

**DOI:** 10.1007/s00213-023-06320-y

**Published:** 2023-02-07

**Authors:** Margaret Moline, Shoji Asakura, Carsten Beuckman, Ishani Landry, Beatrice Setnik, Judy Ashworth, Jack E. Henningfield

**Affiliations:** 1 Eisai Inc., 200 Metro Boulevard, Nutley, Jersey, NJ 07110 USA; 2 Eisai Co., Ltd., Tsukuba, Japan; 3 Formerly Eisai Co., Ltd., Tsukuba, Japan; 4 Formerly Eisai, Nutley, Jersey, NJ USA; 5 Altasciences, Laval, Quebec, Canada and the Department of Pharmacology and Toxicology, University of Toronto, Toronto, Ontario Canada; 6 Pinney Associates, Inc., Bethesda, MD USA; 7 The Johns Hopkins University School of Medicine, Baltimore, MD USA

**Keywords:** Controlled Substances Act, Drug abuse, Abuse potential, Dependence potential, Dual orexin receptor antagonist, Hypnotics, Lemborexant, Rat, Monkey, Human

## Abstract

**Rationale:**

Lemborexant (LEM) is a dual orexin receptor antagonist (DORA) approved in multiple countries including the USA, Japan, Canada, Australia, and several Asian countries for the treatment of insomnia in adults. As a compound with central nervous system activity, it is important to understand the abuse potential of LEM with respect to public health.

**Objectives:**

This review discusses data for LEM relevant to each of the 8 factors of the United States Controlled Substances Act.

**Results:**

LEM did not demonstrate abuse potential in nonclinical testing and was associated with a low incidence of abuse-related adverse events in clinical study participants with insomnia disorder. Similar to other DORAs that have been evaluated (eg., almorexant, suvorexant (SUV), and daridorexant), LEM and the positive controls (zolpidem and SUV) also showed drug liking in a phase 1 abuse potential study that enrolled subjects who used sedatives recreationally. However, internet surveillance of SUV and the FDA Adverse Events Reporting System suggests that drugs in the DORA class display very low abuse-related risks in the community. Additionally, as described in FDA-approved labeling, it does not carry physical dependence and withdrawal risks.

**Conclusions:**

LEM, similar to most other prescription insomnia medications, was placed into Schedule IV. However, LEM and other drugs in the DORA class may have a lower potential for abuse as suggested by real-world postmarketing data from federal surveys and internet surveillance, and thus may have lower risks to public health than Schedule IV benzodiazepines and nonbenzodiazepine hypnotics that potentiate GABA signaling.

## Introduction

Lemborexant (LEM; E2006) is a dual orexin receptor antagonist (DORA) approved in multiple countries, including the USA, Japan, Canada, Australia, and several Asian countries, at doses up to 10 mg for the treatment of adults with insomnia. From the first approval to the end of September 2022, over 100,000 new prescriptions of LEM have been written. Because LEM activity occurs in the central nervous system (CNS), an assessment of its abuse and physical dependence potential was required per Section 21 U.S.C. 811 of the US Controlled Substances Act (CSA). The US Food and Drug Administration (FDA) requires an abuse potential assessment as part of the overall safety assessment of the New Drug Application for CNS-active compounds as the basis for the scheduling recommendation by the drug sponsor as well as the FDA itself (US Food and Drug Administration 2017). The Controlled Substances Act “8-factor analysis” provides an assessment of 8 factors with respect to a drug or other substance proposed to be controlled or removed from the schedules. Factors include the drug’s actual or relative potential for abuse; scientific evidence of its pharmacological effect, if known; the state of current scientific knowledge regarding the drug or other substance; its history and current pattern of abuse; the scope, duration, and significance of abuse; what, if any, risk there is to the public health; its psychic or physiological dependence liability; and whether the substance is an immediate precursor of an already-controlled substance. Further discussion and examples have been previously published (Henningfield et al. 2022), Although the FDA does not require sponsors to include an assessment of the 8 factors of the CSA in their application, it is typically included by sponsors as part of their overall abuse potential assessment to provide the rationale for their scheduling recommendation. For an approvable CNS-active compound, the FDA develops an 8-factor analysis with input by the National Institute on Drug Abuse, which is ultimately provided to the US Drug Enforcement Administration (DEA) by the Assistant Secretary for Health. Since 2016, new drug scheduling actions by the DEA, based on the FDA’s recommendation, have typically occurred within approximately 3 months of the FDA’s notification of approval and the DEA’s receipt of the scheduling recommendation. This process was followed for LEM and the drug was formally placed in Schedule IV of the CSA by the DEA as recommended by the FDA 108 days after FDA approval of LEM on December 19, 2019 (US Drug Enforcement Administration 2020).

Although LEM, similar to most insomnia prescription medications including the DORA SUV, has been classified as a Schedule IV substance, the 8-factor analysis suggests that there are differences between the DORAs and γ-aminobutyric acid (GABA) ergic drugs that may indicate a lower potential for abuse. This review summarizes evidence considered by the FDA and DEA in their scheduling decision and provides some additional data and perspectives gained in the approximate first year since LEM became commercially available.

## Overview of LEM and its development

LEM, as a DORA, is structurally and pharmacologically distinct from other insomnia medications, such as benzodiazepines and the nonbenzodiazepine Z-drugs (zolpidem (ZOL), eszopiclone, zaleplon), which enhance inhibitory GABA signalling. LEM is also structurally and pharmacologically distinct from the melatonin receptor agonist ramelteon and is structurally unique compared with the DORAs SUV and daridorexant (DAR) (Beuckmann et al. 2017; Rappas et al. 2020). The orexin system is a critical upstream modulator of several excitatory signal transduction pathways (Inutsuka and Yamanaka 2013a). The neuropeptides orexin-A and orexin-B are synthesized in neurons of the posterior and lateral hypothalamus; these orexin-expressing neurons project to multiple brain regions, including those involved in the promotion of wake (Scammell et al. 2017; Soya and Sakurai 2020). As a surmountable competitive antagonist, LEM blocks the binding of the orexin-A and orexin-B neuropeptides to their targets, orexin receptor type 1 (OX1R) and orexin receptor type 2 (OX2R). This action reversibly blocks the wake-promoting effects of orexin, thereby reducing wake and promoting sleepiness and sleep (Inutsuka and Yamanaka 2013b; Kärppä et al. 2020b; Rosenberg et al. 2019; Yardley et al. 2021).

In phase 3 studies ranging from 1 month (E2006-G000-304 (Study 304); SUNRISE-1; NCT02783729) to 12 months (E2006-G000-303 (Study 303); NCT02952820; SUNRISE-2) in duration, LEM provided benefit versus placebo on sleep onset and sleep maintenance outcomes, and was generally well tolerated (Kärppä et al. 2020a; Rosenberg et al. 2019; Yardley et al. 2021). Additionally, evidence from these studies and other studies demonstrated that LEM treatment was not associated with next-morning residual effects (Yardley et al. 2021).

### Factor 1: actual or relative potential for abuse

#### Receptor-binding studies

Whereas benzodiazepines and Z-drugs are positive allosteric modulators of the GABA_A_ receptor, LEM is a DORA and therefore has different receptor-binding properties than the GABAergic sleep aids. LEM specifically binds to both OX1R and OX2R, with higher affinity for OX2R (inhibition constant (*K*_i_) = 0.61 nM for hOX2R) than OX1R (*K*_i_ = 4.8 nM for hOX1R), and acts as a competitive antagonist with low nanomolar potency (Beuckmann et al. 2017). In off-target panel binding assays, LEM did not display appreciable binding to 88 potential off-target sites, including other receptors, transporters, and ion channels, aside from the melatonin MT1 receptor (*K*_i_ = 922 nM). Importantly, LEM did not demonstrate off-target binding to opioid receptors, serotonin receptors, central-type and peripheral-type benzodiazepine receptors, dopamine receptors, GABA_A_ receptors, nicotinic acetylcholine receptors, cannabinoid receptors, or noradrenaline, dopamine, and serotonin transporters, all of which are known abuse-related molecular targets (Beuckmann et al. 2017). The M4, M9, and M10 metabolites of LEM bind OX1R and OX2R with similar affinities as LEM, but are P-glycoprotein substrates and are not thought to contribute to biological activity because of their poor brain penetration (Ueno et al. 2021). Mean IC_50_ values against hOX1R were 11.7 nM, 18.6 nM, and 4.2 nM for M4, M9, and M10, respectively; values against hOX2R were 3.8 nM, 4.7 nM, and 2.9 nM (Ueno et al. 2021). These metabolites also did not demonstrate discernible binding to receptors, transporters, or ion channels associated with abuse (Beuckmann et al. 2017).

#### Nonclinical studies in animal models

The abuse potential of LEM was examined in a standard battery of studies based on regulatory guidance, as required for marketing approval. LEM at doses up to 600 mg/kg/day did not produce physical dependence in rats after 28 days of oral dosing as determined via assessment for morning piloerection, salivation, hyperreactivity to handling, tremors and convulsions after being removed from their cages, the presence of loose stools and diarrhea, and other behavioral signs indicative of withdrawal (Asakura et al. 2021). Additionally, in rhesus monkeys who had previously been exposed to multiple drugs, including pentobarbital on a fixed ratio 5 schedule, LEM did not have a positive reinforcing effect (i.e., rhesus monkeys self-administered LEM at rates comparable to that of the vehicle control (Asakura et al. 2021). Similarly, SUV did not have a positive reinforcing effect in rhesus monkeys or produce physical dependence in rats as determined using a functional observation battery (Born et al. 2017). Together, these studies demonstrated that neither DORA had a positive abuse signal in nonclinical testing.

The correspondence between drugs that are self-administered by nonhuman species and those that function as reinforcers in humans has been established (Griffiths and Ator 1980; Griffiths et al. 1980; Griffiths and Johnson 2005; Haney and Spealman 2008; Schuster and Thompson 1969). Although drugs in the DORA class do not have reinforcing effects in animal models, the majority of benzodiazepine and nonbenzodiazepine Z-drugs in Schedule IV maintain self-administration in nonhuman primates, indicative of their positive reinforcing effect. This has been observed for alprazolam, bromazepam, chlordiazepoxide, lorazepam, triazolam (Griffiths et al. 1991), and diazepam (O'Connor et al. 2011), and for ZOL (Griffiths et al. 1992) and zaleplon (Ator 2000). Although benzodiazepines and Z-drugs are included in the same drug schedule, there is a continuum for abuse potential across these compounds. For example, diazepam is considered to have a higher abuse potential than ZOL (Griffiths and Johnson 2005).

In drug discrimination studies in rats, LEM at doses up to 1000 mg/kg did not cross-generalize to ZOL. Partial cross-generalization to ZOL was observed for SUV at higher doses (300 and 1000 mg/kg) but not for lower doses (30 and 100 mg/kg) (Ueno et al. 2021), consistent with a prior study (Born et al. 2017). The highest dose of SUV tested in the prior drug discrimination study, 325 mg/kg, produced a plasma concentration that was approximately 106-fold higher than the maximum plasma drug concentration at the highest approved dose of SUV (Born et al. 2017). In a similar study of LEM, the 1000 mg/kg doses of LEM and SUV produced plasma concentrations that were 118- and 60-fold times the effective human doses of 10 mg and 20 mg, respectively (Ueno et al. 2021). These study results demonstrated a lack of subjective similarity between LEM compared with ZOL or SUV as well as differences between SUV and ZOL. Collectively, the results from nonclinical studies examining physical dependence, self-administration, and drug discrimination indicate that LEM does not demonstrate drug abuse potential. It should be noted that there is now a strong body of preclinical work indicating that drugs of abuse may alter the orexin system, including increasing the number of orexin neurons, which could theoretically alter the action of DORAs. As such, it is important to identify when studies use animals with a prior history of drug exposure and to take this into account when interpreting the findings (Fragale et al. 2021a; James et al. 2019; Thannickal et al. 2018).

#### Human abuse potential study

LEM was examined in the human abuse potential (HAP) study E2006-A001-103 (Study 103; NCT03158025) in comparison with placebo and 2 positive controls, ZOL and SUV (Landry et al. 2022a; Landry et al. 2022b). Study 103 enrolled healthy, nondependent, recreational sedative users who could discriminate both ZOL 30 mg and SUV 40 mg from placebo during a qualification phase. Among subjects who failed qualification (*n* = 107), a greater proportion of those who failed were unable to differentiate SUV from placebo (20/107 (18.7%)) compared with the proportion who were unable to differentiate ZOL from placebo (7/107 (6.5%)). An additional 15 of 107 (14.0%) subjects could not discriminate between either ZOL or SUV and placebo; therefore, overall, 35 of 107 (32.7%) subjects could not discriminate SUV from placebo and 22 of 107 (20.1%) subjects could not discriminate ZOL from placebo. At all doses examined, LEM (10 mg, 20 mg, and 30 mg) demonstrated abuse potential compared with placebo. For the primary endpoint of maximum effect “at this moment” drug-liking, LEM was not statistically significantly different from ZOL or SUV. Overall, this finding was consistent with other secondary endpoints that are collectively used to interpret abuse potential (Landry et al. 2022a; Landry et al. 2022b). The findings from this study are similar to other DORA studies (i.e., almorexant, DAR), that also demonstrated drug-liking (Cruz et al. 2014; Landry et al. 2022b; Ufer et al. 2022).

HAP studies may not always predict real-world abuse potential risk and seem more likely to overestimate rather than to underestimate the risk of abuse, as has been suggested for drugs with novel mechanisms of action that did not demonstrate abuse potential in nonclinical testing (Calderon et al. 2020) or in the community (Caro et al. 2022), suggesting that the HAP studies substantially overestimated the potential for real-world recreational use and abuse in the community. It should also be acknowledged that these HAP studies are routinely conducted with healthy recreational drug users without insomnia to assess the true drug effect and to exclude false negatives. These subjects may not benefit from a DORA. Ratings on scales assessing abuse potential, such as the “at this moment” Drug Liking visual analogue scale, as reported in HAP studies, may be higher in controlled clinical trial settings in an enriched population of recreational users of sedatives than in subjects with insomnia without a predisposition to drug abuse. For example, SUV, which was negative for abuse potential in animal models (Born et al. 2017), was shown to have a similar abuse potential profile to ZOL in a HAP study conducted on recreational polydrug users (Schoedel et al. 2016). However, a review of online discussion threads and posts on drugs-forum.com and Bluelight.org by recreational drug users who shared their experience using SUV did not reveal a trend of recreational abuse of SUV to achieve desired nontherapeutic effects (discussed further in Factor 4). Rather, most online posts discussed its perceived value as an aid to sleep with some comments suggesting that it was of little value for recreational purposes such as getting high. DAR displays similar properties. Although animal studies suggest a lack of abuse potential, a recent study found that DAR displayed dose-related drug-liking among recreational sedative drug users (Roch et al. 2021; Ufer et al. 2022).

This phenomenon has also been observed with other drugs. For example, in a HAP study of the novel opioid analgesic tapentadol, the highest tested dose (75 mg) produced positive ratings on certain measures of abuse potential, similar to what was observed for tramadol and hydromorphone (Stoops et al. 2013). However, as evidenced by postmarketing surveillance data, tapentadol was abused significantly less often than other prescription opioids in the real-world setting (Butler et al. 2015). However, it is important to note that tapentadol has less market penetration compared with other opioids, which may be playing a role in those findings.

Interestingly, diphenhydramine, which is commonly used as an over-the-counter sleep aid, has shown evidence of abuse potential, but is not controlled under the CSA.

On a subjective effects questionnaire, elevated ratings on some measures of abuse potential, including drug liking, end-of-day drug liking, and desire to take the drug again were observed for diphenhydramine (Preston et al. 1992). Scores on some scales with diphenhydramine were comparable to those reported for lorazepam (Schedule IV). Diphenhydramine has also been shown to maintain self-administration in nonhuman primates (Sannerud et al. 1995). These findings suggest that it is important to consider all lines of relevant clinical and nonclinical evidence related to abuse and safety in the evaluation of abuse potential.

#### Abuse-related adverse events in LEM clinical studies in subjects with insomnia

Based on compliance and drug accountability data, there was no evidence of abuse or diversion of study medication (placebo, LEM 5 mg (LEM5), LEM 10 mg (LEM10)) in the 2 pivotal phase 3 LEM clinical studies. Across the phase 3 studies (1-month Study 304 and 12-month Study 303), in which 528, 712, and 705 subjects received at least 1 dose of placebo, LEM5, and LEM10, respectively, a total of 5 overdose events were recorded (1 accidental (LEM10) and 4 intentional (1 placebo, 3 LEM5)). These overdose events were not indicative of abuse potential. No more than 2 tablets were taken instead of 1 in any of these cases. Additionally, none of the intentional overdoses were associated with suicidality or self-injurious behavior, and no treatment-emergent adverse events (TEAEs) were reported in association with these events. It is possible that self-overdosing by patients resulted from their desire for increased efficacy.

Certain adverse events, such as euphoria-related events, have been identified by the FDA as potentially reflective of abuse potential (US Food and Drug Administration 2017). Therefore, the incidence of TEAEs potentially suggestive of abuse potential was examined in a pooled analysis of clinical studies of LEM across the development program conducted in subjects with insomnia disorder (Table 1; *N* = 2488). The most common TEAE related to abuse potential observed in this population was somnolence, which is expected for a sleep-promoting drug (Table 2). Euphoric mood was reported rarely across the LEM clinical studies at the supratherapeutic doses (LEM 15 mg (LEM15) and LEM 25 mg (LEM25)) studied in phase 2, and not at all among subjects treated with therapeutic doses of LEM (LEM5, *n* = 819; LEM10, *n* = 815). After pooling subjects for the LEM15 and LEM25 doses (*n* = 118), euphoric mood was observed at an incidence of 2.5% (3/118). One subject treated with LEM 1 mg in phase 2 study E2006-G000-201 (Study 201; NCT01995838) reported a euphoria-related TEAE (elevated mood). It should be noted that a minority of patients in these pooled analyses received morning administration of lemborexant (instead of night-time), which may contribute to somnolence and potentially reduced or blunted euphoric effects. In subjects with insomnia disorder, incidence rates for “feeling drunk,” dizziness, and hypnagogic hallucinations were also low (< 3%) across LEM groups (Table 2). Other TEAEs related to abuse such as dissociative disorder, feeling abnormal, and emotional disorder were reported in less than 1% of subjects across all LEM doses.

**Table 1:** Overview of LEM clinical studies

Study number	Study design	Dose/treatments	Outcome	*N*^a^	Citation
LEM clinical studies comprising the pooled Safety Analysis Set of subjects with insomnia disorder					
E2006-A001-001 NCT01463098 (Part B)	Two-center, randomized, double-blind, PBO- and active-controlled study	Single oral dose of PBO or LEM (1, 2.5, 5, 10, 25, 50, 100, 200 mg)	Safety and tolerability of single oral doses of LEM	58	Data on file
E2006-A001-107 NCT02350309 (Study 107)	Two-center, randomized, double-blind, PBO-controlled, 3-way crossover study	Single oral dose of PBO, LEM (5, 10 mg), or flurazepam 30 mg (reference drug for assay sensitivity)	Next-morning residual sleepiness and sleep propensity following single oral doses of LEM	69	
E2006-G000-201 NCT01995838 (Study 201)	Multicenter, multiple-dose, randomized, double-blind, PBO-controlled, parallel-group, Bayesian-adaptive, dose-response study	Multiple oral doses of PBO or LEM (1, 2.5, 5, 10, 15, 25 mg)	Identification of a dose or doses of LEM that maximize efficacy and minimize next-day residual sleepiness	291	Murphy et al. (2017)
E2006-G000-303 NCT02952820 (SUNRISE-2; Study 303)	Multicenter, randomized, double-blind, PBO-controlled, parallel-group study	Multiple oral doses of PBO or LEM (5, 10 mg)	Long-term efficacy and safety of LEM compared with PBO	947	Kärppä et al. (2020) Yardley et al. (2021)
E2006-G000-304 NCT02783729 (SUNRISE-1; Study 304)	Multicenter, randomized, double-blind, PBO-controlled, active-comparator, parallel-group study	Multiple oral doses of PBO, LEM (5, 10 mg), or ZOL extended release 6.25 mg	Efficacy and safety of LEM compared with PBO and ZOL	1006	Rosenberg et al. (2019)
LEM clinical studies in healthy subjects without insomnia disorder					
E2006-A001-009 NCT03483636 (Study 009)	Single-center, double-blind, PBO-controlled, single-dose, 4-period crossover, drug-alcohol interaction study	Single oral doses of LEM 10 mg or alcohol (0.7 g/kg for males; 0.6 g/kg for females) alone or in combination	Effects on cognitive performance and postural stability of LEM in combination with alcohol vs LEM alone and vs alcohol alone	32	Landry et al. (2021)
E2006-A001-103 NCT03158025 (Study 103)	Randomized, double-blind, 6-way crossover study	Single oral doses of PBO, ZOL immediate release 30 mg, SUV 40 mg, or LEM (10, 20, 30 mg)	Abuse potential of LEM compared with PBO, ZOL, and SUV	39	Landry et al. (2021)
E2006-E044-106 NCT02583451 (Study 106)	Randomized, double-blind, placebo- and active-controlled, 4-period crossover study	Single oral doses of PBO, LEM (2.5, 5, 10 mg), or zopiclone 7.5 mg (reference drug for assay sensitivity)	Effect of LEM vs PBO on driving performance	48	Vermeeren et al. (2019)
E2006-A001-108 NCT03008447 (Study 108)	Randomized, double-blind, placebo-controlled and active-comparator, 4-period crossover study	Single oral doses of PBO, LEM (5, 10 mg), or ZOL extended release 6.25 mg	Effect of LEM vs PBO and ZOL on postural stability, auditory awakening threshold, and cognitive performance	63	Murphy et al. (2020)

*N*^a ^Refers to the number of subjects who received at least 1 dose of study drug who had at least 1 postdose safety assessment (Safety Analysis Set). *LEM* lemborexant, *PBO* placebo, *SUV* suvorexant, *ZOL* zolpidem

**Table 2:** Summary of abuse-related TEAEs (> 2% in any LEM group) in the pooled Safety Analysis Set from LEM clinical studies in subjects with insomnia disorder

	PBO (*n* = 664)	LEM1–LEM2.5 (*n* = 72)	LEM5 (*n* = 819)	LEM10 (*n* = 815)	LEM15–LEM25 (*n* = 118)
Subjects with any abuse liability TEAE > 2% in any LEM group, *n* (%)					
Somnolence	9 (1.4)	2 (2.8)	51 (6.2)	84 (10.3)	21 (17.8)
Fatigue	1 (0.2)	0	17 (2.1)	18 (2.2)	0
Abnormal dreams	7 (1.1)	2 (2.8)	10 (1.2)	14 (1.7)	0
Nightmare	2 (0.3)	0	8 (1.0)	12 (1.5)	4 (3.4)
Dizziness	13 (2.0)	1 (1.4)	20 (2.4)	10 (1.2)	3 (2.5)
Hypnagogic hallucination	0	0	2 (0.2)	5 (0.6)	3 (2.5)
Feeling drunk	0	0	1 (0.1)	0	3 (2.5)
Euphoric mood	0	1 (1.4)	0	0	3 (2.5)

Subjects who received different treatment during treatment periods were counted under the applicable treatment groups. The pooled Safety Analysis Set included subjects with insomnia disorder from Studies 001 Part B, 107, 201, 303, and 304 as described Table 1. *LEM1*–*LEM2.5*, pooled lemborexant 1 mg and lemborexant 2.5 mg, *LEM5* lemborexant 5 mg, *LEM10* lemborexant 10 mg, *LEM15*–*LEM25* pooled lemborexant 15 mg and lemborexant 25 mg, *PBO* placebo, *TEAE* treatment-emergent adverse event

#### FAERS

When adjusted by the duration of exposure, the overall incidence (subjects per patient-year) of TEAEs related to abuse potential was 0.2 for placebo, 0.3 for LEM5, and 0.4 for LEM10. The overall rates (events per patient-years) of TEAEs related to abuse potential were 0.3 for placebo, 0.5 for LEM5, and 0.6 for LEM10. Moreover, consistent with data from nonclinical studies, evidence from clinical trials in subjects with insomnia disorder indicated that LEM did not produce physical dependence or withdrawal after prolonged use (Yardley et al. 2021). This finding is based on results from the Tyrer Benzodiazepine Withdrawal Symptom Questionnaire (T-BWSQ) (Tyrer et al. 1990), which was administered at the end-of-study visit in Study 201 (data on file), Study 303 (Yardley et al. 2021), and Study 304 (Rosenberg et al. 2019). There was also no evidence of rebound insomnia during the follow-up period of any of these studies (Murphy et al. 2017; Rosenberg et al. 2019; Yardley et al. 2021). Additionally, in the 1-year study (Study 303), the TEAEs reported during the 2-week follow-up period after drug discontinuation did not provide evidence that abrupt discontinuation of LEM produced an acute withdrawal syndrome (data on file). Conversely, Schedule IV benzodiazepines have been shown to produce physical dependence in both animal and human studies (Griffiths and Johnson 2005), a clear differentiation between LEM and most of the other drugs in Schedule IV.

### Factor 2: scientific evidence of the drug’s pharmacologic effects, if known

The pharmacologic activity profile of LEM as observed in nonclinical pharmacology studies and pharmacodynamic evaluations in humans are not suggestive of abuse potential. LEM is absorbed quickly, with a median time to maximum plasma drug concentration (*t*_max_) of 1–3.3 h for the 10-mg oral dose (Landry et al. 2021). In the HAP study (Study 103), scores on the “at this moment” Drug Liking and High Effects visual analogue scales were highest between 1.5–3 h and 1–2 h, respectively, and then declined over time, reaching baseline levels by approximately 8 h postdose. Thus, peak signals of abuse potential in humans coincided with the approximate *t*_max_ of LEM.

The effective half-life (which accounts for drug accumulation and elimination) of LEM is long, with a mean of 17 h for LEM5 and 19 h for LEM10 (Landry et al. 2021). This long effective half-life would be predicted to reduce withdrawal symptoms and repeated self-administration of the drug. Further, the long effective half-life of LEM suggests that if there were withdrawal symptoms after abrupt cessation of chronic use, the symptoms would be relatively weak in intensity and substantially delayed in onset from the time of drug withdrawal compared with a drug with a short half-life. It is generally assumed that within a drug class, a longer half-life, is associated with lower withdrawal. Therefore, withdrawal can be minimized by substitution of one drug with another that has a longer half-life or a formulation and/or drug administration schedule that allows a gradual reduction in plasma levels over time (Brunton LL 2022; Lerner and Klein 2019). However, if the drug is still promoting sleep at wake time, there would be a risk for morning residual effects. The LEM clinical program evaluated this potential extensively and concluded that the risk of such effects was low (Moline et al. 2021b). Thus, it may be possible to avoid rebound, withdrawal and morning residual effects with a longer half-life drug like LEM. In support of this, as reviewed by Lerner and Klein (2019), withdrawal from a benzodiazepine with a 10- to 20-h elimination half-life may have an onset of 1–2 days and last 2–4 weeks compared with an onset of 2–7 days and duration of 2–8 weeks for a benzodiazepine with a > 20-h elimination half-life (Lerner and Klein 2019). Moreover, withdrawal effects were not observed after the discontinuation of LEM after 12 months of treatment (Yardley et al. 2021).

LEM reversibly blocks the binding of orexins to OX1R and OX2R, which in turn inhibits wake-promoting orexin signalling, thereby facilitating sleep. Multiple studies have shown that LEM provides significant benefit versus placebo on sleep outcomes (Kärppä et al. 2020a; Murphy et al. 2017; Rosenberg et al. 2019; Yardley et al. 2021). LEM treatment also reduced the severity of insomnia symptoms and improved daily functioning, as assessed by the Insomnia Severity Index (Rosenberg et al. 2019). Importantly, bedtime dosing provided clinical benefits on sleep without being associated with next-day residual effects (Yardley et al. 2021); these findings will be discussed further in Factor 6.

### Factor 3: the state of current scientific knowledge regarding the drug or other substance

Owing to its complex chemical synthesis and chemical structure, it is unlikely that LEM could be produced by anyone without formal organic synthesis training and significant laboratory resources. LEM is poorly soluble in water and other aqueous solvents. Therefore, although LEM immediate-release tablets could be easily crushed into fine particles using common household items, only small amounts of LEM could be extracted in water, indicating that LEM is not suitable for administration via the injection route. Also, only small amounts of LEM were vaporized when tablets were heated over 250 °C (data on file), suggesting that the inhalation route via smoking is unlikely to be used successfully for drug administration. Abuse via the nasal route is possible; however, it is not known whether LEM is absorbed through the nasal mucosa or if any desired nontherapeutic effects would be produced via insufflation. Thus, if abuse of LEM were to occur, the route of drug administration used would most likely be oral.

The interaction of LEM with alcohol (40% v/v (0.6 g/kg in females and 0.7 g/kg in males]) was examined in a phase 1 study (E2006-A001-009; NCT03483636) (Landry et al. 2022c), which demonstrated that LEM should not be coadminstered with alcohol. Morning alcohol coadministration did not affect LEM *t*_max_ but resulted in a 35% increase in maximum plasma drug concentration and a 70% increase in area under the concentration time curve from time 0 to 72 h compared with LEM alone. LEM alone did not affect postural stability (assessed by body sway), whereas alcohol alone significantly worsened postural stability at 2 h postdose. There was no evidence of an additive effect of LEM with alcohol on postural stability versus alcohol alone. However, additive negative effects of LEM with alcohol on measures of cognitive performance were observed at 2 h postdose, corresponding with the *t*_max_ of LEM; across measures, cognitive performance returned to baseline levels at 6–9 h postdose. No synergy (i.e., more than additive effects) between LEM and alcohol was observed for any pharmacodynamic measure.

As discussed earlier, the orexin system regulates various behavioral and physiological processes, including sleep-wakefulness and motivated drug taking. Interestingly, the orexin system has been shown in several studies to mediate drug-seeking behavior to most drugs of abuse including opioids, cocaine, morphine, and alcohol (James et al. 2021). Correspondingly, there is a wealth of preclinical literature exploring the role of the orexin system on various components of addictive behaviors such as self-administration, craving/drug-seeking, withdrawal, and reinstatement/relapse in substance use disorders such as alcohol, cocaine, and opioids (Baimel et al. 2015; Campbell et al. 2020; James et al. 2021; Perrey and Zhang 2020; Simmons and Gentile 2020; Zarrabian et al. 2020). Investigation into orexin receptor antagonists suggests that this drug class may be an effective therapy for treating substance use disorders in part due to the effects of the orexin pathway on decreasing wakefulness, reward-seeking, and addiction (Fragale et al. 2021b; James and Aston-Jones 2020; Mehr et al. 2021a). While related studies in humans are limited, some have been conducted. One area where orexin receptor antagonists are being investigated therapeutically in humans is in the setting of opioid use disorder. Investigators have hypothesized that an orexin-based approach might directly reduce drug cravings (particularly via actions at Ox1R) and indirectly reduce relapse risk by normalizing sleep disturbances (primarily via Ox2R) (James et al. 2020). Results from a recently published study support this potential as subjects with opioid use disorder demonstrated increased total sleep time and decreased withdrawal symptoms with SUV versus placebo during a buprenorphine/naloxone taper (Huhn et al. 2022). Similar studies, albeit primarily in animals, also suggest a potential therapeutic role for orexin receptor antagonists in cocaine use disorder (James et al. 2021; Simmons and Gentile 2020). Interestingly, a neurobiological link between the orexin system, sleep dysregulation, and food addiction is being investigated towards the potential use of orexin receptor antagonists as a treatment for binge eating disorder (Mehr et al. 2021b). These studies suggest that when administered alongside drugs of abuse, orexin receptor antagonists likely provide beneficial effects in regard to binge eating disorder.

In mice, LEM at oral doses up to 300 mg/kg did not impair motor coordination and balance as assessed by rotarod performance, whereas ZOL 100 mg/kg orally had a strong impairing effect at 1–2 h postdose (Beuckmann et al. 2019). Additionally, LEM did not increase the duration of ethanol-induced anesthesia versus vehicle, whereas ZOL (10 mg/kg or 30 mg/kg orally) significantly increased anesthesia duration versus vehicle (Beuckmann et al. 2019).

In a phase 1 study (E2006-A001-102; NCT03471871), compared with placebo, LEM did not decrease peripheral oxygen saturation or increase apnea-hypopnea index values during sleep after multiple doses (LEM10) in subjects with mild to severe obstructive sleep apnea (Cheng et al. 2020; Moline et al. 2021a) or after a single dose (LEM10 and LEM25) in healthy subjects (Cheng et al. 2021b). Also, no respiratory abnormalities were observed in rat toxicology studies with single oral doses of LEM up to 1000 mg/kg. Together, these studies suggest that LEM is unlikely to cause respiratory depression that could lead to overdose death.

### Factor 4: its history and current patterns of abuse

LEM is only recently marketed in several countries including the USA, Japan, and Canada, and therefore, the history and current patterns of abuse are unknown. Because of the lack of postmarketing data for LEM, insight into potential patterns of abuse was assessed for SUV, which has been marketed in the USA since 2015, by surveillance of popular internet online forums frequented by users of recreational drugs, as described for other pharmaceutical drugs (Cone 2006).

As of September 2022, discussion threads and posts by recreational drug users who shared their experience using SUV (found using the search term “SUV” OR “Belsomra”) were found on the sites drugs-forum.com, Bluelight.org, and Reddit.com. No posts were found on the sites erowid.org, dancesafe.org, or shroomery.org. There was a single mention of LEM in the posts, which referred to LEM as being in the “same class” as SUV. Several hundred postings for SUV and/or Belsomra were identified. The vast majority of posts involved questions about the efficacy for sleep and side effects. A few commented on “recreational value” and “effects.” Typical comments included the following: “Great for insomniacs, but if you’re looking for a recreational drug turn elsewhere” (posted on February 20, 2016) (BLUELIGHT 2016); “If you’re chasing a high, you’ll be disappointed” (posted on July 29, 2016) (reddit 2016); and “from my experience, it has little recreational value” (posted on March 12, 2015) (reddit 2015).

The findings from internet monitoring were consistent with a poster presented by members of the FDA’s Controlled Substance Staff at the June 2020 Annual Meeting of the College on Problems of Drug Dependence. The report focused on drugs with novel mechanisms of action, including SUV, that did not show evidence of abuse potential in animal studies, but were scheduled under the CSA based on results from HAP studies (Calderon et al. 2020).

Several major federal surveys are often considered more definitive and quantitative compared with internet self-report data, but there is typically a 1- to 2-year lag for these data to be published, so at this time, none of them report on LEM. For SUV, there was 1 multi-drug fatality case in a suspected suicide in 2018 reported to the American Association of Poison Control Center’s National Poison Data System. However, there were no mentions of SUV in the 2018 Monitoring the Future Survey nor in the National Forensic Laboratory Information System.

Despite the generally similar abuse potential profile of LEM to SUV and ZOL in the HAP study, postmarketing surveillance will be important to determine if real-world abuse potential is lower than predicted by the HAP study and lower than that associated with benzodiazepine and Z-drug medications. ZOL products were identified as the most misused prescription psychotherapeutic sedative drug listed on the 2019 National Survey on Drug Use and Health among past-year sedative users 12 years or older (National Survey on Drug Use and Health 2019). Similarly, based on an examination of drug abuse–related adverse drug reactions in the European Medicines Agency Database, ZOL was also the most frequently misused/abused Z-drug in Europe (Schifano et al. 2019). Additionally, the FDA recently required updates to the Boxed Warning for drugs in the benzodiazepine class because of concerns related to the risks of these medications for abuse, misuse, addiction, and physical dependence (US Food and Drug Administration 2020). The FDA also issued a Boxed Warning in 2019 for eszopiclone, zaleplon, and ZOL because of rare, but serious, instances of complex sleep behaviors reported in some patients using these medications (US Food and Drug Administration 2019).

### Factor 5: the scope, duration, and significance of abuse

As a recently marketed medication, the scope, duration, and significance of abuse have not been established for LEM. We anticipate that people who use drugs recreationally will try LEM to see whether it produces euphoria and other desirable nontherapeutic effects (i.e., effects not related to improving sleep). Likely oral administration will be tested, and this is expected to produce somnolence but no psychoactive effects such as euphoria (Factor 1). This expectation is based on similar experiences with SUV (Factor 4) and the low incidence of TEAEs related to abuse potential for LEM (Factor 1).

### Factor 6: what, if any, risk is there to the public health

Concerns have been raised over the public health risks associated with some sleep-promoting drugs. For example, many Schedule IV sedative hypnotics, including Z-drug sleep aids, are associated with next-morning residual effects, including negative effects on postural stability and balance (Allain et al. 2005; Mets et al. 2010), cognition (Stranks and Crowe 2014), and driving performance (Gunja 2013). LEM, however, demonstrated sustained efficacy throughout the sleep period without impairment in next-day functioning, as discussed below.

#### Postural stability

ZOL and other Z-drugs are associated with an increased risk of falls and fall-related injuries (Treves et al. 2018). As part of the LEM clinical development program, tests of postural stability (body sway) were conducted to assess fall risk in the middle of the night (approximately 4 h postdose) and in the morning (8 h postdose). Following bedtime dosing, LEM did not impair postural stability in the morning in healthy participants (Study 108; NCT03008447) or in participants with insomnia disorder (Study 304) ≥ 55 years of age (Murphy et al. 2020; Yardley et al. 2021). Conversely, in both studies, subjects treated with ZOL had significantly greater body sway in the morning compared with placebo- or LEM-treated subjects, although this effect was only observed after the first 1–2 nights of treatment, but not after a month of ZOL treatment in Study 304 (Murphy et al. 2020; Yardley et al. 2021). In Study 108, subjects treated with LEM demonstrated greater body sway than placebo in the middle of the night, but significantly less than with ZOL (Murphy et al. 2020).

#### Cognitive function

The effect of LEM versus placebo and versus ZOL was assessed using a Cognitive Assessment Performance Battery (Murphy et al. 2020; Yardley et al. 2021). In Study 108, there was no impact on the performance of any tests of attention or memory in the middle of the night for healthy participants receiving LEM5. However, middle-of-the-night performance was worse on these tasks for subjects receiving LEM10 and ZOL when compared with placebo (Murphy et al. 2020). Morning assessments from Study 108 revealed no differences between LEM versus placebo or ZOL versus placebo on the tests of attention and memory. In morning assessments from Study 304, tests of memory and continuity of attention (vigilance) upon awakening did not show differences between LEM and placebo. However, in Study 304, the power of attention (ability to focus attention and process information) was significantly faster with placebo than LEM or ZOL, and there were no significant differences between LEM and ZOL (Yardley et al. 2021).

#### Driving performance

A concern associated with the use of sleep-promoting drugs is the potential to impair driving ability in the morning after bedtime use because of residual effects (Gunja 2013; Vermeeren 2004). Therefore, next-day driving performance is important in assessing the public health risk of LEM relative to other sleep aids, as several of the currently marketed Schedule IV sedative hypnotics including ZOL have been shown to impair driving performance (Gunja 2013) and increase the overall risk of driving accidents and increase the risk of vehicular accident responsibility (Hansen et al. 2015; Orriols et al. 2011).

Morning driving performance was not impaired in an on-road driving study and no morning driving tests were prematurely stopped after single or multiple evening doses of LEM (Vermeeren et al. 2019). For SUV, there was no significant impairment in overall next-morning driving performance, as assessed by mean changes in the standard deviation of lateral position (Vermeeren et al. 2015; Vermeeren et al. 2016). However, a symmetry analysis revealed that significantly more SUV-treated subjects than placebo-treated subjects 23–64 years of age had changes in the standard deviation of lateral position values that were indicative of impairment, and 5 driving tests were stopped prematurely (2 subjects at 40 mg, 1 subject at 20 mg, and 1 subject at 20 mg and 40 mg) due to subjective drowsiness (Vermeeren et al. 2015). For DAR, a statistically significant impairment in next-morning simulated driving performance was reported in both adult (50–64 years of age, median = 58 years) and elderly (65–79 years of age, median = 70 years) subjects versus placebo, after a single night-time administration of either a 50 mg or 100 mg dose. After 4 consecutive nights of DAR administration with either 50 mg or 100 mg, the mean effect on driving performance was not statistically significant versus placebo, although impaired driving ability was reported in some subjects receiving DAR (Muehlan et al. 2022). Although no studies to date have reported an association between LEM use and driving accident risk, a potentially increased risk cannot be excluded.

#### Additional considerations

Across LEM studies in subjects with insomnia, abuse potential–related TEAEs linked to psychomotor and cognitive challenges were very low: balance disorder (0.2% placebo, 0.1% LEM5, and 0.2% LEM10), cognitive disorder (0.2% placebo, 0.1% LEM5, and 0% LEM10), and disturbance in attention (0.2% placebo, 0% LEM5, and 0.4% LEM10). Additionally, in daily sleep diary assessments of morning alertness, subjects rated themselves as more alert after treatment with LEM than with placebo (Yardley et al. 2021). There were no clinically important sex-, race-, or age-related differences in measurements of next-day residual sleepiness (Landry et al. 2021).

In the LEM HAP study, all doses of LEM, which were administered in the morning, were associated with subjective feelings of drowsiness. However, in cognitive assessments, LEM was associated with faster speed of motor response and of processing information, better motor precision, and better divided attention capabilities compared with placebo and ZOL (Landry et al. 2022a).

As discussed in Factor 3, no synergy between LEM and alcohol was observed on cognition or postural stability. However, additive negative effects were observed on some cognitive measures for LEM with alcohol at 2 h postdose, but these effects resolved over time. The theoretical potential for pharmacodynamic interactions between LEM and other drugs causing sedative effects (e.g., sedatives, hypnotics) remains. One theoretical concern for any sleep-promoting drug, including DORAs, is that coadministration with respiratory depressant drugs such as opioids, could lead to the blockade of brainstem orexin neurons that directly innervate central autonomic and respiratory regions. Accordingly, one might consider the potential precipitation or exacerbation of sleep-disordered breathing or other respiratory complications that are commonly observed in opioid use disorder (James et al. 2020). It should be noted that respiratory safety studies with LEM and other DORAs did not cause clinically meaningful effects on the apnea-hypopnea index or peripheral oxygen saturation after single and multiple doses (Boof et al. 2021a; Boof et al. 2021b; Cheng et al. 2020; Cheng et al. 2021a; Sun et al. 2016; Uemura et al. 2015). Until more is known about potential synergistic effects of LEM with other drugs, coadministration of alcohol or other CNS depressants with LEM should be avoided.

Overall, for subjects treated with LEM, there was less impairment of posture, motor function, cognition, and residual morning effects that are risk factors for falls or motor vehicle accidents relative to currently marketed Schedule IV sleep aids.

### Factor 7: its psychic or physiologic dependence potential

In a study conducted in rats, there was no evidence of withdrawal symptoms suggesting that LEM does not produce physical dependence in rats (Asakura et al. 2021). Consistent with this finding, as discussed in Factor 1, the T-BWSQ administered at the end of the follow-up period of phase 2 (data on file) and phase 3 clinical trials (Rosenberg et al. 2019; Yardley et al. 2021) identified no evidence of withdrawal symptoms for LEM compared with placebo. There was also no evidence of rebound insomnia or of an acute withdrawal syndrome after discontinuation of LEM.

Similar to LEM, SUV did not produce physical dependence in rats as determined using a functional observation battery (Born et al. 2017). In contrast, as described in Factor 1, benzodiazepines and nonbenzodiazepine Z-drugs have been shown to produce physical dependence in both animal and human studies.

### Factor 8: whether the substance is an immediate precursor of a substance already controlled

LEM is not structurally related to any controlled drug of abuse, nor do its starting material, intermediates, and major metabolites serve as chemical precursors to any known controlled substance.

## Conclusions

All lines of relevant clinical and nonclinical evidence related to abuse and safety are considered in the 8-factor evaluation of drug abuse potential. Overall, the totality of evidence suggests that LEM may be less likely to be abused and associated with overdose in the real-world setting compared with GABA-ergic drugs approved for insomnia. This is supported by differences in its mechanism of action, pharmacology, and abuse potential as discussed in this review. LEM did not produce a positive abuse potential signal in nonclinical testing and did not produce withdrawal symptoms upon acute discontinuation in phase 3 clinical studies. The incidence of abuse-related TEAEs such as euphoria was low across the LEM clinical program. Additionally, LEM provided significant benefit on sleep and was not associated with next-morning residual effects.

Similar to findings with other DORAs (e.g., almorexant, DAR, SUV), LEM showed abuse potential in a phase 1 HAP study, and the similarity in abuse potential signals with ZOL and SUV was important in the FDA’s and DEA’s determination that placement in Schedule IV was appropriate for LEM (DEA, 2020). We agree that this placement was appropriate without the benefit of postmarketing data for LEM. However, as discussed in Factors 4, 5, and 6, postmarketing data from federal surveys and internet surveillance for SUV, another drug in this orexin inhibitor class of drugs suggest relatively lower real-world abuse, overdose, and other safety risks for this drug class compared with benzodiazepine sedative hypnotics.

Postmarketing evidence from ongoing safety surveillance will be important to determine how real-world rates of abuse and/or problematic use of LEM compare to those observed with benzodiazepines and other sedative hypnotics. If postmarketing data and clinical studies demonstrate that LEM is an insomnia treatment that provides sustained sleep efficacy, reduced next-day functioning impairment and fewer risks of injury than other Schedule IV sleep aids, then less restrictive scheduling may merit consideration in the interests of patient safety and public health. Indeed, less restrictive scheduling may lead to increased consideration and access to LEM and potentially other DORAs by patients and physicians for insomnia treatment. This is particularly important for patients who may be more susceptible to adverse effects of Schedule IV sleep-promoting agents (e.g., elderly). The availability of another sleep-promoting pharmacologic may provide a treatment option for patients who may be at risk.
